# Bisphosphonates and Atypical Femur Fractures: Is the Relationship Causal or Casual?

**DOI:** 10.7759/cureus.48141

**Published:** 2023-11-02

**Authors:** Rajeshwaree Bal, Ratnakar Ambade, Nihaal Singh, Prateek Upadhyay

**Affiliations:** 1 Medicine, Jawaharlal Nehru Medical College, Datta Meghe Institute of Higher Education and Research, Wardha, IND; 2 Orthopaedics, Jawaharlal Nehru Medical College, Datta Meghe Institute of Higher Education and Research, Wardha, IND

**Keywords:** bone mineral density, metabolic bone disease, femur fractures, atypical fractures, bisphosphonates

## Abstract

Bisphosphonates (BPs) are a time-tested drug class with multivariate use cases. They are used in pathologies ranging from osteoporosis to Paget's disease, and also help in accelerated fracture healing. They have been used to treat both benign and malignant lesions of the skeletal system since a long time. However, there have been reports of increased incidences of atypical femoral fractures (AFFs) in patients exhibiting chronic use of bisphosphonates in the past years. This has led to the widespread dissuasion of physicians and practitioners from using the drug class. By means of this review of the literature, the authors aim to investigate the relationship between BP use and its association with AFFs. The review focuses on and elucidates the basic pharmacology of BPs and goes on to illustrate the indications of BPs in various pathologies of the musculoskeletal system, further exploring the effects of BPs on the healing of various bony fractures. The authors also explore the incidences of other pathologies, such as osteonecrosis of the jaw and nephropathies associated with BP use, and elaborate on their features. Through this review, the authors have tried to educate and induce critical thinking on the part of clinicians and medical professionals in regard to prescribing BPs to patients that need them, by keeping in mind the risk-reward relationship that accompanies their use.

## Introduction and background

The most frequently prescribed medication for common skeletal illnesses such as osteoporosis, metastatic bone disease, and Paget's disease of the bone is bisphosphonates (BPs). BPs have distinct pharmacological characteristics that set them apart from other treatments, such as selective skeleton uptake that occurs primarily at locations with enhanced bone remodeling and slow release from bone [1].

Osteoporosis has some of the most incapacitating effects of aging with features of bone loss and microarchitectural degradation [2,3]. Osteoporotic fractures are becoming more common and more expensive as the world population is aging. The diagnosis and treatment of those who are most at risk are crucial to prevent fractures and their repercussions, as many fractures result in significant impairment and disease-related fatality [4,5].

In individuals with osteoporosis, bisphosphonates lessen the likelihood of vertebrae-related and non-vertebrae-related fractures and stop bone volume reduction, but because BPs limit the bone turnover rate by inhibiting osteoclast function and inducing apoptosis, BP therapy is linked to negative side effects [6].

## Review

Pharmacology of bisphosphonates

For the best therapeutic results and to reduce the risk of negative effects, it is crucial to understand BPs' pharmacology as well as the variations between the different members of the class. In theory, studying pharmacokinetics and pharmacodynamics can improve clinical decision making and lead to the selection of a specific BP, route of administration, and dose in the individual patient. However, in reality, the data that have been released thus far are not the best, are frequently difficult to analyze, and at times do not provide sufficient evidence for the conclusions that have been made [1]. They are structurally stable counterparts of pyrophosphate and so have a high affinity for bone mineral. By reducing the frequency of activation and the rate of remodeling, they slow down the osteoclasts' capacity to reabsorb bone. Unambiguous proof that the incidence of fractures has decreased can be found in large prospective randomized placebo-control trials. The safety profile of bisphosphonates has been equally comforting for 40 years since their first usage in patients, which is impressive [7].

Indications of BP therapy

Bisphosphonates have been the cornerstone of osteoporosis therapy ever since they were first introduced in the 1990s. Bone remodeling and resorption caused by osteoclasts are inhibited by bisphosphonates [2]. BPs have been shown to be effective in increasing bone mineral density (BMD) and lowering the risk of hip and vertebral fractures by as much as 40% to 70% in numerous large, randomized, controlled trials [4].

For many years, osteoporosis, multiple myeloma, Paget's disease, and other conditions characterized by low bone mineral density have all been treated with BPs, which are also known as phosphonates. Bisphosphonates have been extensively researched in preclinical models for their impact on fracture healing because they prevent bone resorption, a crucial stage of fracture repair [8].

The American Society for Bone and Mineral Research (ASBMR) task team published a list of traits displayed by patients with AFFs; 92.3% of the patients used BPs to treat osteoporosis, while only a small percentage used them to treat cancer. Majority of the subjects were women, and were rather younger than those who had standard osteoporosis-related fractures of the femur. The average time spent on BP medication regime was seven years. About 70% of subjects experienced prodromic pain in the thigh or groin. In 28% of the subjects, there were bilateral complete fractures and radiological irregularities, including cortical responses; 26% of patients experienced delayed healing [6].

Effect of bisphosphonates on fracture healing

BPs are effective against osteoporotic changes and promote accelerated healing of fractures. Low bone volume and bone quality degeneration are symptoms of osteoporosis, which raises the likelihood of low-energy fractures. By inhibiting bone resorption, BP therapy helps osteoporosis patients build more bone volume and lower their chance of fracture. In individuals suffering from osteoporosis, BPs lessen the likelihood of vertebrae-related and non-vertebral fractures and reduce bone volume reduction [6].

In the case of high-resolution x-ray structures of the human enzyme in complexes with risedronate and zoledronate, two of the most popular nitrogen-containing BPs (N-BPs) in clinical use, these substances cause a conformational shift by attaching to the ligand pockets specific to dimethylallyl or geranyl pyrophosphate. The N-BP cyclic nitrogen type drug interactions seen in association with Thr-201 and Lys-200 indicate that these blockers become productive by placing their nitrogen in the said carbocation-binding region [9].

Hip and osteoporotic fractures can be reduced with the use of bisphosphonates, but the prevalence of hip fractures may be rising as a result of concerns about atypical femoral fractures (AFFs), which have significantly reduced the usage of bisphosphonates. There are still significant questions about the relationship between bisphosphonates and other risk factors for atypical femur fractures [2,3].

Although there is no delay in the creation of the fracture callus, BP treatment in animal models is linked to a bigger fracture callus and a delay in the remodeling from primary woven bone to lamellar bone [8]. The de novo use of BP medication following fracture does not seem to significantly affect fracture healing in people. Rarely, patients who use bisphosphonates for long term may experience an atypical fracture and a delay in fracture healing [9].

Atypical femoral fractures

Odvina and colleagues provided the initial description of AFFs in 2005. They hypothesized that prolonged BP medication would cause an oversuppression of bone remodeling, impairing the body's capacity to heal skeletal microcracks and causing skeletal fragility to rise. The task force of the ASBMR outlined all primary and secondary characteristics pertaining to these fractures and summarized the published reports on AFFs [3].

**Figure 1:**
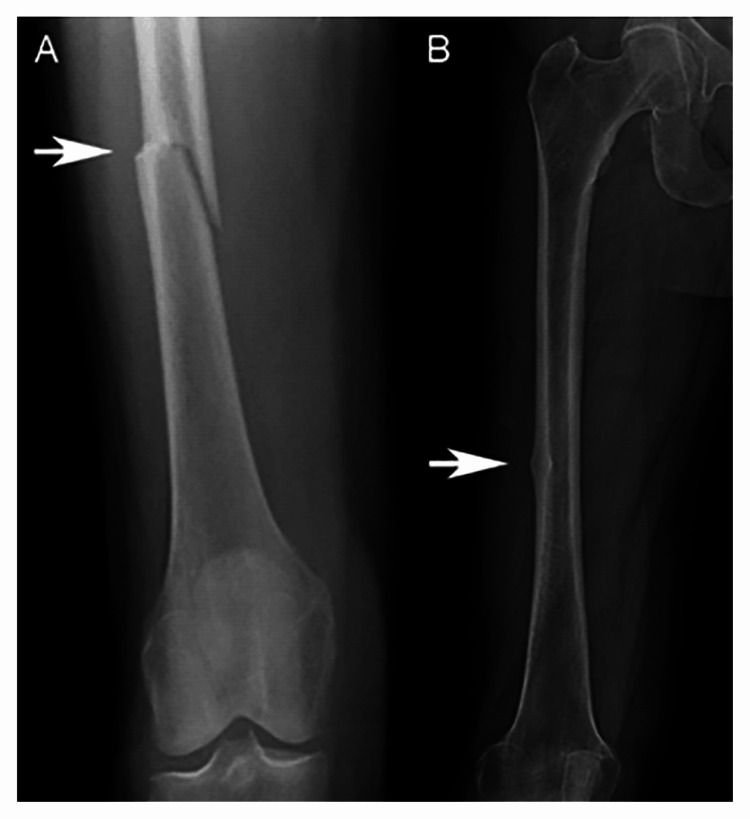
Radiographic views of an (A) atypical femoral fracture that occurred spontaneously in a woman after six years of treatment with a bisphosphonate and (B) incomplete atypical femoral fracture with cortical thickening that presented as thigh pain. Source: Khan AA, Kaiser S. Atypical femoral fracture. CMAJ. 2017, 89:E542 Open access journal under a CC-BY license

AFFs are distinguished from stress fractures or responses by distinctive radiographic (transverse line of the fracture, formation of periosteal callus at the site of fracture, negligible comminution) and quantifiable (prodromic pain, both sides) aspects. Based on fresh data, the ASBMR updated the first case description to emphasize uniqueness of radiological characteristics that show AFFs are different than regular diaphyseal fractures of the femur related to osteoporosis and to give more detailed explanations of what transverse orientation actually entails [9]. Figure 1 shows a radiographical image of complete and incomplete femoral fractures.

Bisphosphonates have recently come under scrutiny for their potential role in atypical fractures, an uncommon form of minimally traumatic or atraumatic femur fracture that occurs below the great trochanter. Calls for a more thorough investigation of the lasting outcomes of bisphosphonate therapy have been sparked by this query. The Food and Drug Administration failed in its attempt to establish agreement and offer clear opinions. This has caused ongoing anxiety in both patients and treating doctors, which has contributed to a general decline in the number of prescriptions for BPs and other osteoporosis treatments [4].

According to the results of a matched case-control study by Lenart et al., postmenopausal women who don't have any clear secondary causes of bone loss could develop low-energy subtrochanteric/shaft fractures after taking bisphosphonates for an extended period of time. Additionally, long-term bisphosphonate use was linked to a specific type of femur fracture, known as a simple transverse or oblique fracture with cortical thickening and beaking in the subtrochanteric/shaft region. This fracture is unusual for osteoporotic females [10].

When the interfragmentary distance is insufficient and the strain is excessive in situations of osteoclast dysfunction, preventing the physiologic healing process from occurring. Therefore, the continuation of this dysfunction could result in a stress fracture that is clinically visible [11,12].

In the femur, which is abundant in cortical bone and biologically designed to bear strong, repeating stresses, spontaneous or low-trauma fractures are uncommon [11]. Although antiresorptive medicines boost bone mineral content, long-term exposure may affect the characteristics of the cortical bone material, which could be harmful to bone strength. By drug class, the effects could differ [12]. Alendronate was connected to lower modulus loss at failure (lessened tendency for a material to bend) and reduced fatigue life (fewer cycles of stress before failure) in a four-point bending study of femurs from osteoporotic sheep exposed to raloxifene, alendronate, zoledronate, or teriparatide for a year [13,14].

Stress and pathological fractures, hypophosphatasia and brittle bone disease are other diseases that can be confused with atypical femoral fractures. Looser zones can be mistaken for AFFs but typically are findings seen medially, and can be brought on by hypophosphatemic osteomalacia. Although a femoral stress fracture may develop below the lesser trochanter, it is typically seen medially, and not on the lateral side of the cortex [15].

Other pathologies where BPs have been implicated

Osteonecrosis of the Jaw

Osteonecrosis of the jaw (ONJ) is characterized by necrosis in the jaw bone that is exposed, pain, potential supplementary infection, enlarged, tender lesions, and different dysesthesias; however, not many such severe instances go unnoticed [16].

There is a concern regarding BP-related ONJ (BRONJ), in the field of dentistry. Because of their high binding to bone hydroxyapatite and non-hydrolyzable P-C-P structure, BPs accumulate in bones after repeated administration [17]. BPs are taken up by osteoclasts during bone resorption and exhibit cytotoxicity, generating a long-lasting anti-bone-resorptive action. N-BPs and non-N-BPs are two categories of BPs. N-BPs are responsible for BRONJ and have much stronger anti-bone resorptive actions than non-N-BPs [13].

Osteoclasts die through starvation when bisphosphonates, which do not include nitrogen, are converted into an inactive, toxic analogue of adenosine triphosphate. By blocking farnesyl diphosphate synthase in the mevalonate pathway, second-generation bisphosphonates, which include nitrogen, prevent osteoclast vesicular trafficking, membrane ruffling, morphology, and cytoskeletal organization [12].

Bisphosphonate Nephropathies

Since the 1980s, bisphosphonate nephropathy has been documented, and in the 1990s, more research was done to determine whether this class of drugs has any renal tolerability. Year 2008 saw a thorough assessment of the research on the toxic effects of pamidronate, zoledronate, and ibandronate on the renal system. Nevertheless, new details have recently come to light. Additionally, there are now more bisphosphonates on the market, including alendronate, risedronate, and clodronate [18].

The majority of the research on bisphosphonate nephrotoxicity discusses neoplasm-affected patients who may be more vulnerable to the nephrotoxic effects of a high cumulative dose of bisphosphonates administered over a shorter period of time as well as the concomitant use of other nephrotoxic medications, like cytostatic therapy. Most individuals, but not all, appear to be able to reverse the adverse renal effects of bisphosphonates. While zoledronate has primarily been linked to direct tubular toxic effects, pamidronate has been linked to nephropathy based on focal segmental glomerulosclerosis (FSGS) [18].

In conclusion, primarily zoledronate and pamidronate may cause an impairing in renal function. The new discovery that 40% of cancer patients continue nephrotoxic medications despite an impaired renal function emphasizes the significance of being aware of the potential nephrotoxic effects of bisphosphonates and the significance of measuring and monitoring renal function when prescribing these medications [19].

Medical management of atypical femur fractures

Teriparatide, denosumab, raloxifene are some of the most recommended drugs for AFFs, but each has been associated with some degree of variability in healing and favorable results. The minimal observational data suggest that teriparatide may promote quicker healing in surgically treated AFFs. When bone turnover markers (BTMs) are high in postmenopausal women who do not have a history of venous thromboembolic events, raloxifene may be explored as a follow-up after teriparatide medication [20,21].

The immediate cessation of bisphosphonates is necessary if an incomplete fracture is found. Sufficient calcium and vitamin D should be given to patients. To avoid the progression to a complete fracture, orthopedic intervention with intramedullary nailing is advised if there is pain. If there is no pain, therapy can be modest and weight-bearing should be restricted for two to three months. Prophylactic nailing should be considered if there is no improvement [22].

Patients with significant thigh pain and those with a radiolucent fracture line on radiographs should be advised to undergo prophylactic intramedullary nailing to reduce the risk of complete fracture and improve functional and clinical outcomes; however, non-operative treatment of AFFs may be tried in patients without it [23].

After three to five years of continuous use, the need for antiresorptive pharmacological therapy should be reevaluated. Patients with AFF risk factors and those with a lower 10-year fracture risk may benefit from taking a "drug holiday" from their osteoporosis therapy [24].

“Drug holiday” regimen and its effects

Drug holiday is a guideline by the ASBMR task force, where patients on bisphosphonates are given a period of temporary “holiday/break” from therapy, which has shown some evidence of lowering risks of further AFFs; also, drug efficacy may be retained [17]. Many ongoing trials are still trying to decipher the same. A drug holiday may continue with yearly monitoring if the patient has a low risk of fractures [25]. Depending on the patient's BMD/BTM and fracture status, the drug holiday can range from two to five years if they have a moderate to high risk of fractures [26].

Each patient should have an individually tailored drug holiday that lasts for a specific amount of time. Serial bone mass measures, bone turnover rates, and an analysis of the patient's fracture history should all be used in the evaluation [27]. Only poor BMD and advanced age at the time of cessation were linked with off-therapy fracture in the Fracture Intervention Trial Long-term Extension (FLEX) study after five years of alendronate treatment. Similar risk variables, such as prior fractures and their recentness, were present in the Health Outcomes and Reduced Incidence with Zoledronic acid Once Yearly - Pivotal Fracture Trial (HORIZON-PFT) extension study after zoledronic acid withdrawal. Therefore, younger people (aged 65-75) who have successfully completed three to five years of BP treatment without fracture and whose BMD is no longer in the osteoporotic range (T-score > 2.5) are the best candidates for starting a drug holiday [28,29]. Although short-term changes in BMD and BTM were not linked to fracture risk in the FLEX study, older age and poor BMD at the time of termination were [30].

## Conclusions

With the help of this review, the authors want to communicate the various use cases of BPs, gauge their relationship with AFFs and eliminate any misconceptions about their efficacy and safety. The authors urge the clinicians and the practitioners to make informed decisions regarding prescribing and administering BPs to patients that need them. In clinical practice, it is of utmost importance that the patient’s safety and well-being is kept at the forefront of the decision-making process. This review of the literature explores BPs in terms of their pharmacodynamics and pharmacokinetics, along with their role in fracture healing and managing osteoporotic changes in the musculoskeletal system. The review also elaborates on the effects of BPs on inducing AFFs, which have been a trend of late. There is also some emphasis on some other pathologies that may see BPs as contributory factors, mainly osteonecrosis of the jaw and BP-related nephropathies. The authors strongly believe that this narrative review will provide a scaffolding on which foundations of better clinical decisions can be constructed.
